# Association Between Immunosuppressive Cell Populations and Immune Response to Influenza Vaccination in Younger and Older Adults

**DOI:** 10.3390/vaccines14080680

**Published:** 2026-08-07

**Authors:** Daniela Di Placido, Antonio Ciaramella, Antonella Riccomi, Claudia Maria Trombetta, Maria Dorrucci, Francesca Farchi, Roberto Giuseppetti, Serena Marchi, Nunzia Sanarico, Patrizio Pezzotti, Anna Rita Ciccaglione, Emanuele Montomoli, Catia Valdarchi, Silvia Vendetti

**Affiliations:** 1 Department of Infectious Diseases, Istituto Superiore di Sanità, 00169 Rome, Italy; 2 Department of Molecular and Development Medicine, University of Siena, 53100 Siena, Italy; 3 Center for Control and Evaluation of Medicines, Istituto Superiore di Sanità, 00169 Rome, Italy; 4 VisMederi Research srl, 53100 Siena, Italy

**Keywords:** influenza vaccine, immunoregulatory cells, MDSCs, Treg, immune response to vaccination

## Abstract

Background: Seasonal influenza vaccination shows highly variable effectiveness across individuals, particularly in older adults, in whom age-related immune decline compromises protective responses. Methods: This study evaluated immune responses to the 2019/2020 quadrivalent influenza vaccine in 75 individuals aged 25–89 years. Influenza-specific antibody titers and virus-neutralizing activity were measured before and four weeks after vaccination, and participants were stratified as high or low responders. Results: We observed that older individuals exhibited increased frequencies of myeloid-derived suppressor cells (MDSCs) and activated regulatory T cells (Treg) expressing HLA-DR and CD38, consistent with enhanced immunosuppressive activity. Importantly, higher levels of these immunoregulatory populations correlated with poor vaccine responsiveness in both adult and older participants. Additionally, circulating T follicular regulatory (Tfr) cells were elevated in older participants and in low responders, whereas T follicular helper (Tfh) cells remained unchanged, resulting in an increased Tfr/Tfh *ratio* associated with impaired antibody production. However, when adjusting for possible confounders, most immunoregulatory subsets did not remain independently associated with responder status, except CD38^+^ Treg cells. Conclusions: These findings suggest that immunoregulatory networks, while essential for controlling chronic inflammation during aging, may limit vaccine-induced immunity. Identifying biomarkers such as MDSC frequency, activated Treg phenotype, and Tfr/Tfh balance may contribute to the identification of individuals with different levels of vaccine responsiveness and could help inform future personalized vaccination strategies.

## 1. Introduction

Vaccination plays a pivotal role in protecting individuals against infectious diseases, yet the effectiveness of vaccines can vary significantly across different age groups. Variability in vaccine efficacy among individuals represents a well-established characteristic across multiple vaccine platforms and age groups, including both younger and older populations [1]. Predicting who will respond optimally to a given vaccine is a critical aspect of vaccine development [2]. Notably, the older population faces distinct challenges in mounting robust immune responses to vaccination compared to younger individuals [3]. This phenomenon is attributed to several key factors that collectively contribute to diminished vaccine effectiveness in aging populations. Firstly, advancing age is associated with a natural decline in immune function, a process known as immunosenescence [4,5]. This gradual deterioration compromises the elderly’s ability to mount effective immune responses following vaccination, leading to lower levels of protective immunity compared to their younger counterparts. Furthermore, variability in vaccine responsiveness among older individuals underscores the influence of diverse factors such as baseline immunological profile, comorbidities, and genetic differences [3,6,7]. Identifying individuals with suboptimal vaccine responses is imperative for tailoring vaccination strategies to enhance efficacy. With advancing age, a chronic low-grade inflammation called inflammaging develops [8,9]. Chronic inflammation needs to be balanced by anti-inflammatory mechanisms to prevent inflammation from destroying tissue integrity. Cells with immunoregulatory properties such as regulatory T lymphocytes (Treg) or myeloid-derived suppressor cells (MDSCs) are immunosuppressive cells that are induced and recruited by pro-inflammatory cytokines to inhibit inflammatory reactions, promote the resolution of inflammation, and initiate the process of repair [10,11,12,13]. Although much has been studied regarding tumors and their responses to infectious diseases, there is evidence indicating that MDSCs increase with age in mouse models [14,15]. An increase in MDSCs during aging was also observed in humans, with many review studies suggesting an increase in MDSCs with age [12,16,17,18] and limited papers showing such an increase [19,20]. Therefore, these observations would be of interest for further evaluation on a higher number of individuals. Furthermore, the exact role of these populations in regulating the immune response in older individuals is not clear yet.

In this study, we analyzed the response to the influenza vaccination in older donors compared to adults and analyzed host factors that could be implicated in a lower responsiveness. We explored the hypothesis that cells with immunoregulatory properties have an inverse correlation with the ability to respond to influenza vaccination.

## 2. Materials and Methods

### 2.1. Cohort Description and Sampling

A cohort study of donors was recruited in 2019–2020 (*N* = 75), between October 2019 and January 2020, by their general practitioners located inRome municipality, Lazio region, Italy. Study participants were classified into three age groups according to [21]: young adults (>25 and <50 years), adults (≥50 and <65), and older adults (≥65). Participants were vaccinated with the quadrivalent influenza vaccine (QIV) Vaxigrip (Sanofi Pasteur, Lyon France), which contained hemagglutinin from the following viral strains: an A/Brisbane/02/2018 (H1N1) pdm09-like virus (Clade 6B.1A1); an A/Kansas/14/2017 (H3N2)-like virus (Clade 3C.3a); a B/Colorado/06/2017-like virus (B/Victoria/2/87 lineage) (Subclade 1A_Δ2); and a B/Phuket/3073/2013-like virus (B/Yamagata/16/88 lineage) (Clade 3) [22]. Participants diagnosed with cancer, immunological disorders, receiving immunosuppressive therapy, subjected to transplant, suffering from viral or bacterial infections, pregnant women, and subjects to vaccinations in the last 6 months were excluded from the study. Participants with chronic diseases such as cardiac diseases, diabetes, asthma, and COPD were included in the study, and the presence of the pathologic condition was evaluated as a possible confounder during the analysis. In addition, all the participants had received an anti-influenza vaccination at least once before. They were invited to complete a questionnaire assessing frailty according to Rockwood’s scale [23], while the evaluation of frailty was carried out by means of the handgrip strength test [24].

Blood was drawn from participants before the anti-influenza vaccination and 28 days after. Following vaccine administration, we tracked the humoral immune responses against the influenza antigens included in the vaccine.

### 2.2. Ethics Statement

Ethical approval for the study was obtained from the Ethics Committee of the Istituto Superiore di Sanità (A00-ISS 30/05/2019 0016650). Research activities were conducted in accordance with relevant regulatory requirements and internationally recognized ethical guidelines, including the International Conference on Harmonisation (ICH) Guideline for Good Clinical Practice (GCP) and the ethical principles derived from the Declaration of Helsinki. Before any study-related procedures were performed, participants signed their written informed consent, which included the acceptance of sampling procedures and the collection and management of data for scientific purposes. All records were subsequently anonymized to protect participant privacy.

### 2.3. Serum Samples

Serum samples were collected anonymously and handled in compliance with Italian ethical regulations. Sheep hyperimmune antisera supplied by the National Institute for Biological Standards and Control (NIBSC) were included as positive controls: A/Brisbane/02/2018-like (H1N1, 19/102), A/Kansas/14/2017-like (H3N2, 19/152), B/Colorado/06/2017-like (B/Victoria lineage, 18/170), and B/Phuket/3073/2013 (B/Yamagata lineage, 15/150). Human serum lacking IgA, IgM, and IgG immunoglobulins (Sigma-Aldrich, S5393) was used as the negative control.

### 2.4. Hemagglutination Inhibition Assay

A hemagglutination inhibition (HI) assay was conducted according to a previously published protocol [25], with minor modifications. Before analysis, serum samples, including sheep hyperimmune sera and negative controls, were treated with a receptor-destroying enzyme (RDE) derived from *Vibrio cholerae* (Sigma-Aldrich, Milan, Italy) at a 1:5 *ratio* and incubated at 37 °C for 18 h. Samples were subsequently heat-treated at 56 °C for 60 min in the presence of 8% sodium citrate (1:4 dilution). Fresh turkey erythrocytes were washed twice by centrifugation in 0.9% saline and prepared as a 0.35% suspension. Serum samples were initially diluted at 1:10 and then serially diluted two-fold in duplicate in 96-well microplates using physiological saline. Standardized influenza antigen containing 4 hemagglutinating units (HAU) in 25 μL was added to each well, followed by incubation at room temperature for 1 h. Subsequently, turkey red blood cells were added, and the plates were incubated for an additional hour before visual assessment of hemagglutination inhibition. HI titers were determined as the reciprocal of the highest serum dilution capable of completely preventing hemagglutination. Since assay testing began at a dilution of 1:10, antibody titers below this limit were considered undetectable and were assigned a value of 5 for statistical analyses.

### 2.5. Influenza Virus Micro-Neutralization Assay

The influenza virus micro-neutralization assay was carried out according to a previously published protocol [25] with some modifications. MDCK (Madin–Darby Canine Kidney) cells were cultured in Eagle’s minimum essential medium (EMEM) supplemented with 2 mmol/L L-glutamine, 10% fetal bovine serum (FBS), 1% non-essential amino acids, and 100 U/mL penicillin-streptomycin. Cells were maintained at 37 °C in a humidified atmosphere containing 5% CO_2_ and were used up to passage 30. Prior to analysis, serum samples were heat-treated at 56 °C for 30 min and subsequently subjected to two-fold serial dilutions in EMEM supplemented with 0.5% FBS on a 96-well plate. Diluted sera were combined with an equal volume of viral suspension corresponding to 100 TCID_50_ per well and incubated for 1 h at 37 °C under 5% CO_2_. Following this step, MDCK cells were added at a final concentration of 1.5 × 10^5^ cells/mL, and plates were incubated for an additional 5 days under standard culture conditions (37 °C, 5% CO_2_). At the end of the incubation period, viral cytopathic effects (CPE) were assessed by light microscopy. Wells displaying CPE in more than 50% of the cell monolayer were considered positive for infection. Neutralizing antibody titers were defined as the reciprocal of the highest serum dilution capable of preventing CPE.

### 2.6. Flow Cytometry

Peripheral blood mononuclear cells (PBMCs) were isolated from the participants through density gradient centrifugation, stained, and analyzed by flow cytometry. Cells were incubated for 20 min at 4 °C with the following anti-human fluorochrome-conjugated antibodies: HLA-DR (clone AC122, Miltenyi Biotech), CD45 (clone 2D1), Lin (CD3 clone UCHT1, CD4 clone RMA-T4, CD8, clone RMA-T8, CD19 clone SJ25C1, CD56 clone MEM-188), CD33 (clone WM53), CD11b (clone IMRF44), CD14 (clone 61D3), CD15 (clone MEM-158) all from Immunological Sciences, Rome, Italy. Following staining, cells were washed (10 min, 1500 rpm, RT) and resuspended in FACS buffer prior to acquisition. The subclasses of MDSCs were identified among the Lin^−^ HLA-DR^low/−^ CD11b^+^ CD33^+^. Moreover, the expression of CD15 and CD14 was used to distinguish polymorphonuclear MDSCs (PMN-MDSCs) and monocytic MDSCs (M-MDSCs), respectively. PMN-MDSC detection by flow cytometry in PBMC samples was performed according to the previously published protocol [26]. Whole blood samples were analyzed immediately after collection to determine the phenotype of immune cell populations. Samples were stained using the following anti-human fluorochrome-conjugated antibodies: CD127 (clone MB15-18C9), HLA-DR (clone AC122) from Miltenyi Biotech, Bergisch Gladbach, Germany; CD45 (clone 2D1), CD4 (clone RMA-T4), CD8 (clone RMA-T8), all from Immunological Sciences; and CD25 (clone M-A251), CD38 (clone HIT2), CD45RO (clone UCHL1), CXCR5 (clone RF832), all from Becton Dickinson Franklin Lakes, NJ, USA. T regulatory cells were identified as CD45^+^CD4^+^CD25^+^CD127^low^ cells, consistent with the phenotype originally described to identify suppressive T cells [27], and their activation state by HLA-DR and CD38 expression [28,29]. Peripheral T follicular helper cells and peripheral T follicular regulatory cells were identified as CD45^+^CD4^+^CD45RO^+^CXCR5^+^ and CD45^+^CD4^+^CD45RO^+^CXCR5^+^CD25^+^ cells, respectively. Whole blood was stained with fluorochrome-conjugated mABs for 10 min, lysed with lysing solution (Becton Dickinson) for 10 min, washed twice with FACS buffer (1% FBS/PBS), and acquired on CytoFLEX LX (Beckman Coulter, Brea CA, USA) or Gallios (Beckman Coulter). Appropriate unstained and FMO controls were performed during the experimental setup, and after doublet exclusion, CD45^+^ cells were further analyzed following the gate strategy described below. Samples were analyzed by Kaluza Analysis Software version 2.3 (Beckman Coulter).

### 2.7. Statistical Analysis

Characteristics of the cohort were examined across three age groups (>25–<50, ≥50–≤65, and ≥65 years). Categorical data were described using absolute and relative frequencies, whereas continuous data were summarized by their median and corresponding interquartile range (IQR). We evaluated the relationship between two continuous variables by simple linear regression, i.e., assessing whether the relative beta coefficient was statistically different from zero. To assess the effect of potential confounders, a multivariable regression model was used. Statistical comparisons between two independent groups were conducted using the non-parametric Mann–Whitney U test. Vaccine responsiveness was evaluated through a stratification approach based on HI antibody titers measured 28 days after immunization. Participants were grouped according to the number of strain-specific responses exceeding the strain-specific median, resulting in five categories (0–4). Subjects displaying titers above the median for zero or one strain were classified as low responders (LR), whereas those exhibiting titers above the median for two or more strains were considered high responders (HR), as previously described [30]. This classification does not represent a validated clinical threshold. To assess the association of HR (vs. LR) with both age and baseline titer, we applied logistic models with HR (vs. LR) as the outcome and age and ln baseline titer (both as continuous) as the independent variables. Logistic models were first applied separately according to specific antigens; thus, the statistical unit was the individual. Secondly, we applied a unique model, particularly a multiple logistic mixed model with HR (vs. LR) as the outcome and with ln-transformed baseline titer, age, and antigen type as independent variables, allowing for dependence of titers on the same subject. To assess the possible association between age and low responsiveness, we applied a multivariable logistic regression model using LR vs. HR as the dependent variable and age, BMI, comorbidities, and frailty as independent variables. Odds ratios (ORs) and 95% confidence intervals (95% CIs) were estimated. To control for multiple comparisons, Benjamini–Hochberg false discovery rate (FDR) correction was applied. All reported associations remained statistically significant after FDR adjustment (adjusted *p* values < 0.05); therefore, nominal *p* values are reported. We used GraphPad Prism version 10.6.1 (GraphPad Software, Boston, MA, USA) and SAS/STAT version 9.4 (SAS Institute, Cary, NC, USA) for statistical analyses.

## 3. Results

### 3.1. Characteristics of the Study Population

In 2019–2020, 75 participants were enrolled in the study. They were classified into three age groups: young adults (>25 and <50 years, n = 15), adults (≥50 and <65 years, n = 23), and older adults (≥65, n = 37). Out of 75, 43 (57.3%) were females, and 32 (42.7%) were males (Table 1), with a higher proportion of females than males in the young adult (73.3% vs. 26.7%) and adult categories (60.9% vs. 39.1%), and with a slightly lower proportion of females in older participant category (48.6% vs. 51.4%). The median age was 41 years (range: 27–49 years; interquartile range: 37–43 years) in the young adult group, 57 years (range: 52–65 years; interquartile range: 55–58 years) in the adult group, and 76 years in the older adult group (range: 66–89 years; interquartile range: 71–79 years). Overall, participants had experienced higher (48%) or middle/lower (52%) education, with a higher proportion of higher education among the young adult (73.3 vs. 26.7) and adult (73.9 vs. 26.1) participants and a lower proportion of higher education in the older adults (21.6% vs. 78.4%). Regarding body mass index (BMI), 50.7% had a normal weight, 34.2% were overweight, and 13.7% were obese, with a higher proportion of normal-weighted individuals in the young adult group (80%) and progressively less in the adult group (52.2%) and even less in the older participant group (37.1%). Smoking at the time of vaccination was declared by 13.3% of participants, with a similar proportion among the three age groups. Most of the study participants (57.3%) showed medical conditions during the current season. Of those, the highest percentage was found among the elderly population (78.4%), whereas fewer were observed in adults (39.1%) and even fewer in young adults (33.3%), which was as expected. The most frequent diseases found in the study population are chronic cardiac diseases (45.3%), diabetes (12%), asthma (8%), and COPD (9.3%). About one-third of patients (32%) reported one comorbidity, and 25.0% reported more than one comorbidity (i.e., asthma, heart disease, and diabetes). Regarding frailty, the majority (65.3%) of study participants declared to be fit (score 1–2, according to Rockwood’s scale [23]) at the time of enrolment, and 34.7% were vulnerable/mildly frail (score > 2), with a higher proportion of frail participants among the older adults (45.9%). Frailty was evaluated by assessing the handgrip strength [24] (in 61 participants out of 75) of the participants, and the lower values were found in the older population (48.1%), and progressively higher values were found in adults (63.6%) and even more in young adults (91.7%). Participants in the study received the seasonal influenza vaccine and were bled both before and after 28 days. We tracked their humoral immune responses against the influenza antigens included in the vaccine. Since the SARS-CoV-2 virus started to circulate in the region at the time of the sample collection, all sera were tested before and after receiving flu vaccination, and they were also tested for anti-Nucleoprotein and anti-Spike antibodies, all of which were negative.

### 3.2. Circulating Blood Myeloid-Derived Suppressor Cells Increase with Age

**Figure 1:**
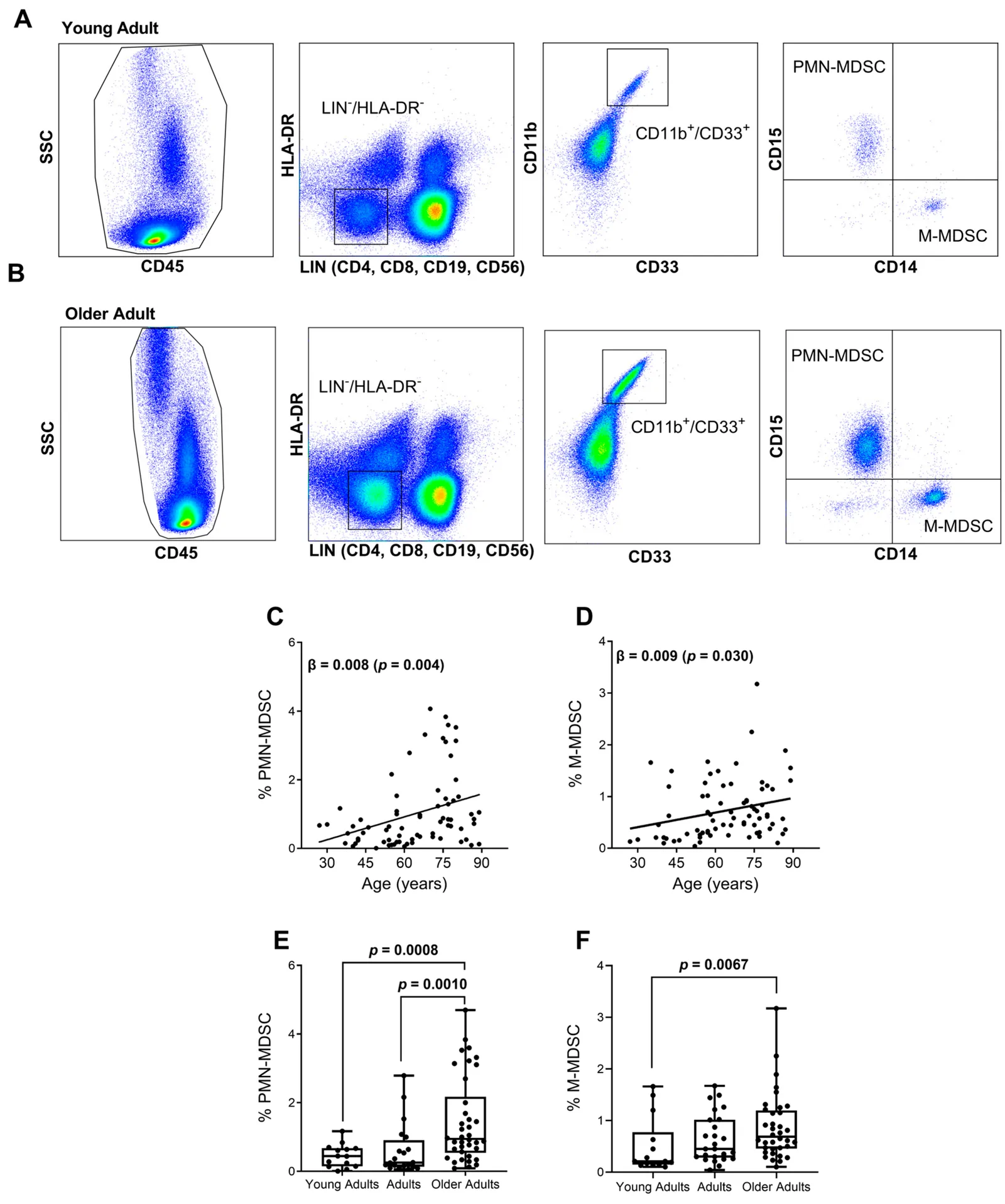
PMN-MDSCs and M-MDSCs are increased with age. The subclasses of MDSCs were identified among the Lin^−^HLA-DR^low/−^CD11b^+^CD33^+^, and the expression of CD15 and CD14 was used to distinguish PMN-MDSCs and M-MDSCs, respectively. Representative plots of the gating strategy in (**A**) young (aged >25 and <50 years) and (**B**) older adults (aged >65 years) are shown. A significant positive association was observed between the increase in age and the percentage of both PMN-MDSCs ((**C**), n = 72) and M-MDSCs ((**D**), n = 75). The percentage of PMN-MDSCs ((**E**), n = 72) and M-MDSCs ((**F**), n = 75) in older adults (>65 years) as compared with adults (≥50 and ≤65) and young adults (>25 and <50) is shown. Linear regression analysis was applied to evaluate the association between percentages of PMN-and M-MDSCs and age, and a non-parametric Mann–Whitney test was applied to evaluate the differences between the groups.

The percentage of MDSCs in the participants enrolled in the study was analyzed in young adults, adults, and older adults. Peripheral blood mononuclear cells (PBMCs) were isolated from the participants and analyzed by flow cytometry. The subclasses of MDSCs were identified among the Lin^−^HLA-DR^low/−^CD11b^+^CD33^+^. Additionally, the expression of CD15 and CD14 was used to distinguish polymorphonuclear MDSCs (PMN-MDSCs) and monocytic MDSCs (M-MDSCs), respectively. Representative plots of the gating strategy in a young and older donor are shown in Figure 1A,B. A significant positive association was observed between the age of donors and the percentage of both PMN-MDSCs (β = 0.008, *p* = 0.004) (Figure 1C) and M-MDSCs (β = 0.009, *p* = 0.03) (Figure 1D). The association was also significant in the adjustment for BMI, comorbidity, and frailty (PMN-MDSCs: β = 0.027, *p* = 0.006; M-MDSCs: β = 0.011, and *p* = 0.017). Moreover, a significant increase in the percentage of the PMN-MDSCs was observed in the older adults as compared with the adults (0.95 (0.54–2.18) vs. 0.26 (0.14–0.91), *p* = 0.0010) (Figure 1E) and young adults (0.95 (0.54–2.18) vs. 0.45 (0.15–0.68), *p* = 0.0008) (Figure 1E). Progressive increase in M-MDSCs with age has been observed, and differences between older and young adults were significant (0.69 (0.46–1.20) vs. 0.21 (0.15–0.77), *p* = 0.0067). These data confirm that MDSCs increase with age in humans, suggesting that they could be induced by the low grade of inflammation described in the elderly.

### 3.3. Activated CD4^+^CD25^+^ T Regulatory Cells Increase with Age in Peripheral Blood

**Figure 2:**
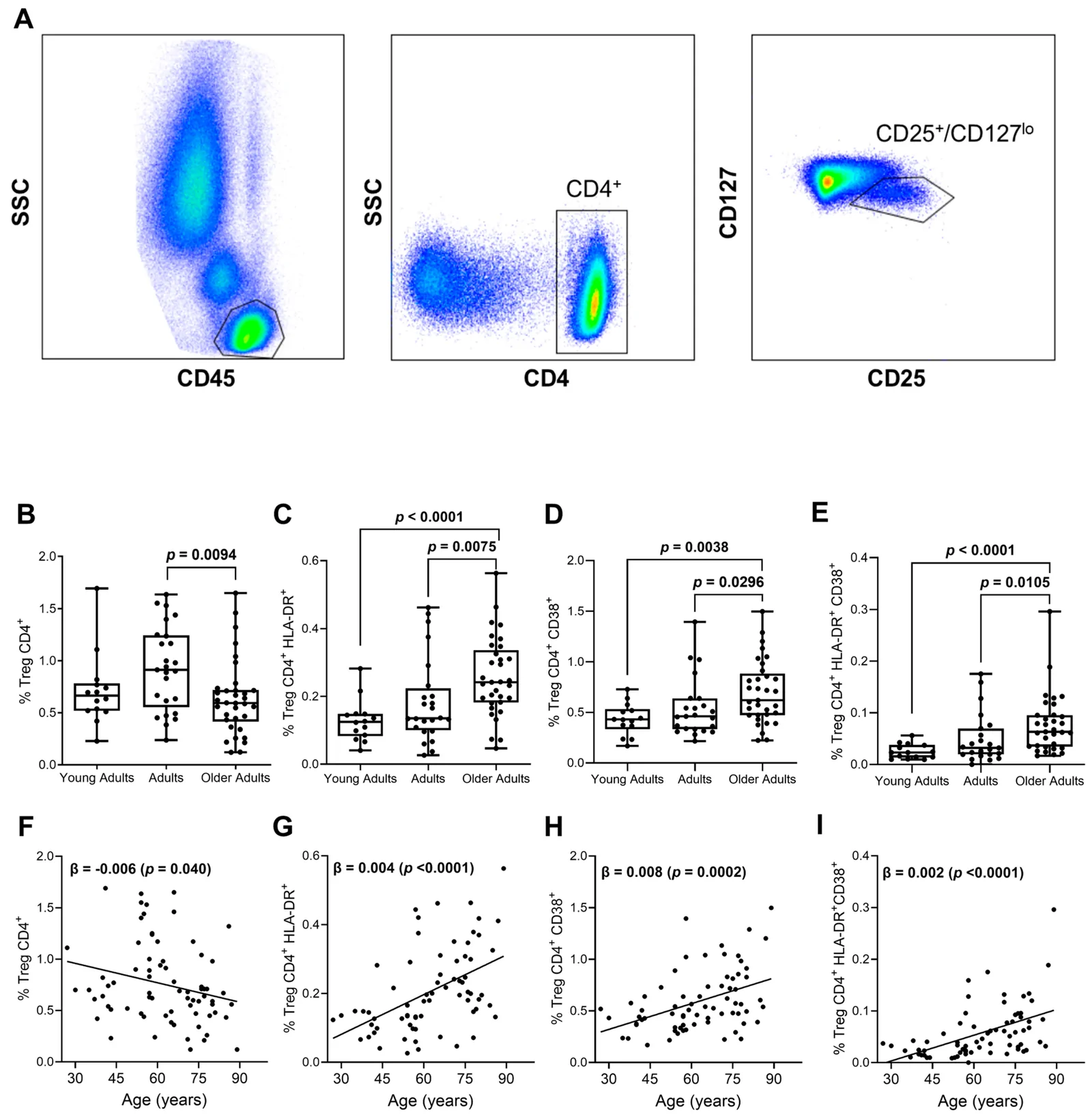
Percentages of CD4^+^CD25^+^CD127^low^ Treg cells across different age groups. (**A**) Representative plots of the gating strategy are shown. (**B**) The percentage of Treg CD4^+^ cells (CD4^+^ CD25^+^CD127^low^) calculated relative to the CD45^+^ cells (n = 73), (**C**) the percentage of Treg CD4^+^ HLA-DR^+^ (n = 71), (**D**) CD4^+^CD38^+^ (n = 70) and (**E**) CD4^+^HLA-DR^+^ and CD38^+^ (n = 70) all estimated relative to the CD4^+^ cells, across individuals belonging to the three groups analyzed are shown. A significant positive association was observed between age and the percentage of (**F**) Treg CD4^+^, (**G**) Treg CD4^+^HLA-DR^+^, (**H**) Treg CD4^+^CD38^+^, and (**I**) Treg CD4^+^HLA-DR^+^ and CD38^+^. Linear regression analysis was applied to evaluate the association between percentages of Treg subpopulations and age, and a non-parametric Mann–Whitney test was applied to evaluate the differences between the groups.

The percentage of T regulatory cells belonging to the CD4^+^CD25^+^ Treg was evaluated in the peripheral blood of the study participants. Cells were identified by cytofluorimetric staining of whole blood as CD45^+^CD4^+^CD25^+^CD127^low^, as shown in the representative plot in Figure 2A. The percentage of CD4^+^CD25^+^ Treg was either not different between young and older adults or was significantly lower in older adults as compared with adult participants (0.59 (0.42–0.72) vs. 0.91 (0.55–1.24), *p* = 0.0094). Analysis of activation markers on Treg revealed that the older adults exhibited a significantly higher proportion of Treg expressing HLA-DR than adults (0.24 (0.18–0.34) vs. 0.14 (0.10–0.22), *p* = 0.0075) and even more than young adults (0.24 (0.18–0.34) vs. 0.13 (0.08–0.15), *p* < 0.0001; Figure 2C). Similarly, CD38 expression was significantly higher in older individuals than in adults (0.62 (0.47–0.88) vs. 0.46 (0.34–0.64), *p* = 0.0296) and young adults (0.62 (0.47–0.88) vs. 0.43 (0.33–0.53), *p* = 0.0038; Figure 2D). Finally, the percentage of Treg expressing both the HLA-DR and CD38 activation markers was higher in the older individuals than in the adults (0.06 (0.03–0.10) vs. 0.03 (0.02–0.07), *p* = 0.0105) and young adults (0.06 (0.03–0.10) vs. 0.02 (0.01–0.04, *p* < 0.0001; Figure 2E). In addition, linear regression analysis between the CD4^+^CD25^+^ Treg cell subpopulations and age showed that Treg cells decrease with age (β = −0.006, *p* = 0.040; Figure 2F), whereas the percentages of activated Treg subpopulation either expressing HLA-DR (β = 0.004, *p* < 0.0001; Figure 2G) or CD38 (β = 0.008, *p* = 0.0002; Figure 2H) or both (β = 0.002, *p* < 0.0001; Figure 2I) significantly increased with age. The association was calculated also adjusting for BMI, comorbidity and frailty and the results were consistent with the unadjusted analysis of the activated Treg cell subpopulations (Treg cells: β = −0.004, *p* = 0.260; TregCD4^+^HLA-DR^+^ β = 0.004, *p* < 0.001; Treg CD4^+^CD38^+^ β = 0.009, *p* < 0.001; and Treg CD4^+^HLA-DR^+^ CD38^+^ β = 0.002, *p* < 0.001). These data indicate that in the older participants, Tregs show an activated phenotype and are present in a higher percentage, suggesting that they can play a role in modulating the immune responses to the vaccinations.

### 3.4. Qualitative and Quantitative Analysis of the Immune Responses to Seasonal 2019/2020 Influenza Vaccine

The 2019/2020 quadrivalent influenza vaccine was administered to all enrolled subjects according to the recommendations of the Italian health authorities. To monitor the effects of vaccination on the immune system, peripheral blood samples were collected before immunization and at day 28 following vaccine administration. The hemagglutination-inhibition (HI) antibody titer was evaluated in the sera of the study participants at baseline (D0) and at D28 post-vaccination.

**Figure 3:**
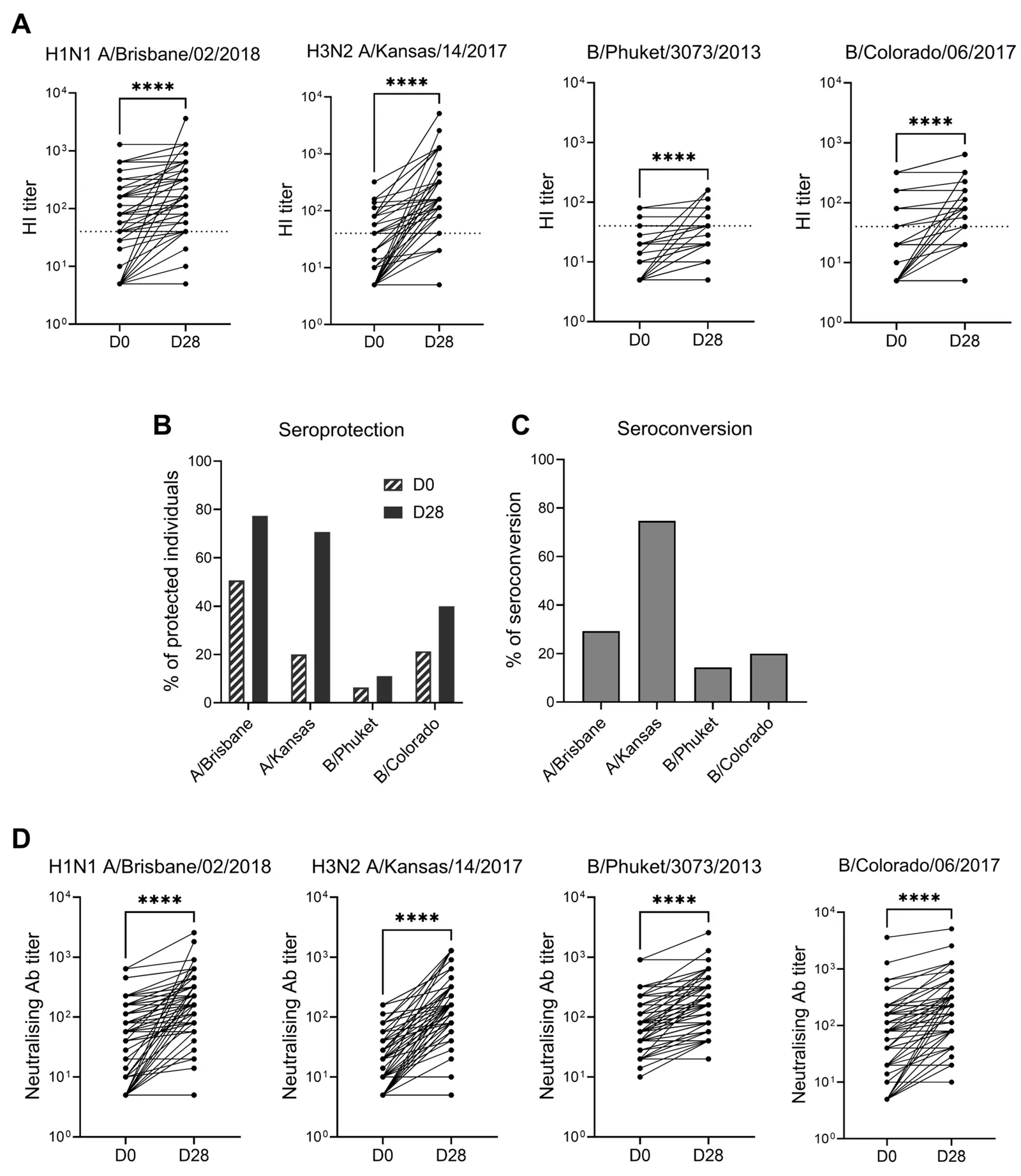
Humoral immune response to influenza vaccination. (**A**) Basal (D0) HI titers and the response at day 28 (D28) in vaccinees for each one of the influenza virus strains after vaccination (n = 75). Seroprotection of subjects is indicated by the dotted line for HI titers ≥40. Percentage of (**B**) seroprotection in vaccinees at baseline (D0) and day 28 (D28) and (**C**) seroconversion for each one of the four influenza virus strains. (**D**) Results from the microneutralization assays performed on the same donors. The neutralization capacity is expressed as the reciprocal of the highest dilution of the donors’ serum at which virus infection is blocked. Each line connects paired measurements from the same participant. All paired comparisons between D0 and D28 are shown in panels (**A**,**D**) and were statistically significant (Wilcoxon matched-pairs signed-rank test, **** *p* < 0.0001).

**Figure 4:**
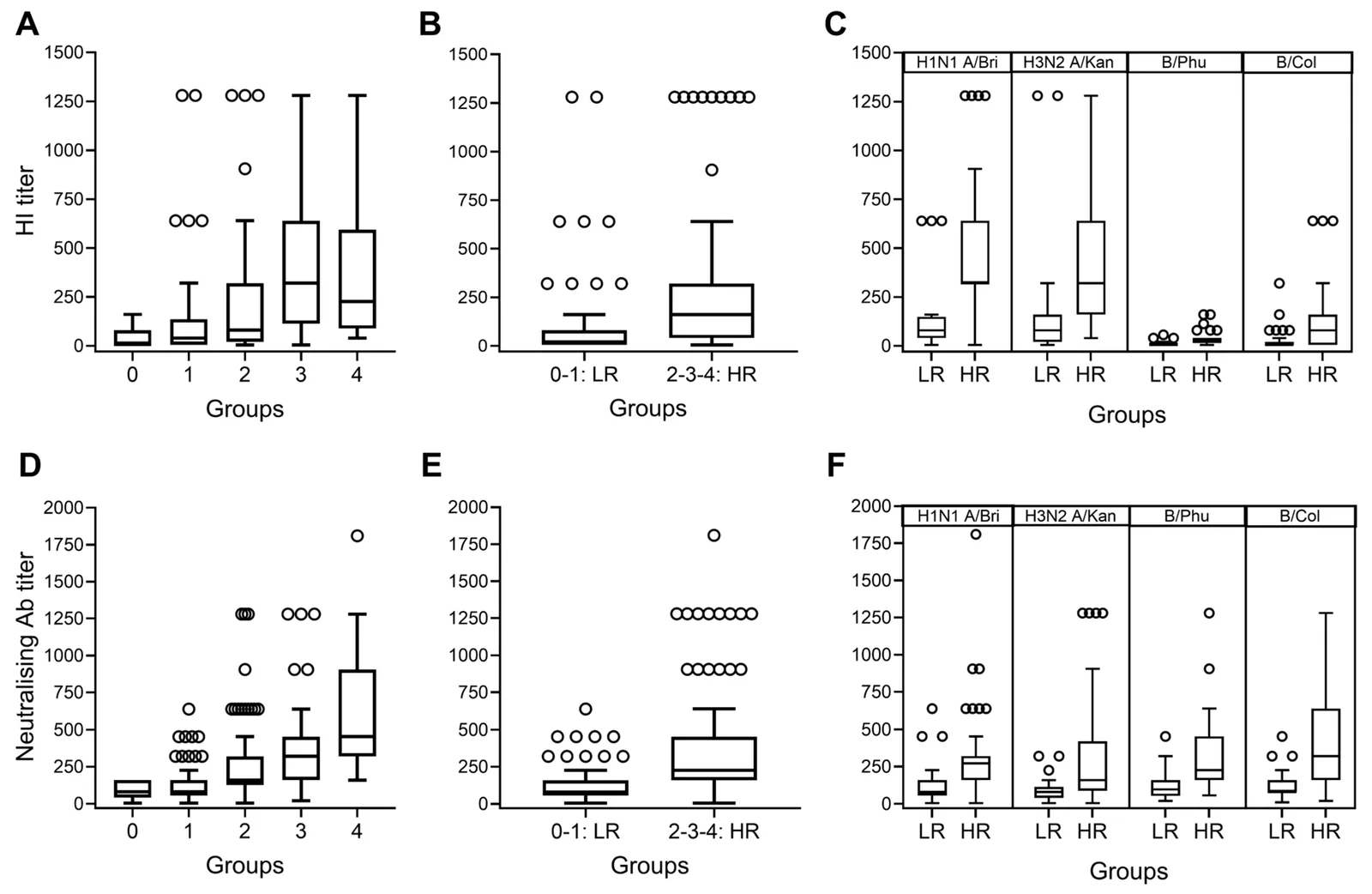
Stratification of the subjects based on the response to the four influenza virus strains. The study population was stratified in five groups considering as a reference the median of (**A**–**C**) HI titer and (**D**–**F**) the microneutralization (MN) titer of each post vaccination specific viral antigen: (**A**,**D**) individuals with no specific HI or MN titer above the specific median post vaccination (D28) (0) and individuals with one (1), two (2), three (3) and four (4) specific HI or MN titers above the specific median at D28 post vaccination, respectively. (**B**,**E**) Participants belonging to group 0 or 1 were classified as low responders (LR), and participants belonging to groups 2, 3, or 4 were classified as high responders (HR). The differences between LR and HR were statistically significant (*p* < 0.001). (**C**,**F**) Classification in HR and LR was statistically significant for each viral strain included in the vaccine. Boxplots summarize antigen-specific observations obtained for the four vaccine strains. Individual dots correspond to antigen-specific observations (n = 300) and not to individual participants; dots outside the whiskers represent outliers.

The influenza-specific antibodies produced 28 days following vaccination against the four antigens included in the vaccine showed a significant increase in HI titers (Figure 3A; Wilcoxon matched-pairs signed-rank test, *p* < 0.0001). Seroconversion, according to international guidelines, is defined as a >4-fold increase in HI titers over baseline and seroprotection as HI titers ≥40. Not all participants reached seroconversion, and a high number of them were seroprotected before receiving the vaccine. Differences between the percentages of protection at baseline were observed for the four viral strains, and the highest percentage of protection was found for the A/Brisbane strain (50%). Overall, the percentage of protection 28 days post-vaccination increased for all viral strains included in the vaccines, even if to different extents (Figure 3B). The high baseline titers observed could be a result of previous exposure to the influenza virus or to earlier vaccinations. On the other hand, the percentage of seroconversion was low among the viral antigens except for the H3N2 A/Kansas, which reached about 70% of seroconversion (Figure 3C). In addition, to verify whether the antibodies against the viral antigens were functional in preventing viral infection, a microneutralization (MN) assay was performed by incubating the sera with the four different viruses, and their virus neutralization capacity was evaluated. Antibodies elicited by influenza vaccination were functional, as the virus neutralization capacity was significantly increased, which is consistent with the HI results (Figure 3D; Wilcoxon matched-pairs signed-rank test, *p* < 0.0001). Given the pronounced inter-individual variability observed in vaccine-induced responses, participants were categorized according to the strength of their humoral response, following an approach previously described [30]. For each vaccine strain, the median post-vaccination (D28) HI titer was used as a threshold. Subjects were stratified into five groups: those with a strain-specific HI titer below the post vaccination specific median value at D28 (0) and those with one (1), two (2), three (3), and four (4) strain-specific HI titers above the specific median value at D28 (Figure 4A). Individuals belonging to groups 0 and 1 were designated as low responders (LR), whereas those classified in groups 2, 3, and 4 were classified as high responders (HR) (Figure 4B); this classification does not represent a validated clinical threshold. In addition, when considering the high-response definition, we assessed that there is a high frequency of HR events also adjusting for baseline values and age: we applied multiple logistic models according to the four antigens, finding OR > 1 adjusting for H1N1 ln-baseline and ln-B/Colorado (*p* = 0.005 and *p* < 0.0001), while the *p*-value was 0.607 adjusted by H3N2 ln-baseline and 0.082 adjusted for ln-B/Phukets. In addition, we applied a multiple logistic mixed model with HR (vs. LR) as the outcome and with ln-(baseline titer), age, and antigen-type as independent variables, and only age was statistically significantly associated with HR (OR = 0.94 for one age-year increment; 95% CI: 0.89–0.99; *p* = 0.031). The same groups were identified according to the analysis of the MN titers, and the population was stratified into five groups (Figure 4D), grouped in HR and LR (Figure 4E), and analyzed for each viral strain (Figure 4F). The numbers of participants in the LR and HR groups were 41 (55%) and 34 (45%), respectively. In the LR group, 22 (54%) were female, and 19 (46%) were male; in the HR group, 21 (62%) were female, and 13 (38%) were male. The median age was 71 years (range: 30–89 years; interquartile range: 57–77 years) in the LR group and 57 years in the HR group (range, 27–86 years; interquartile range, 50–70 years).

**Figure 5:**
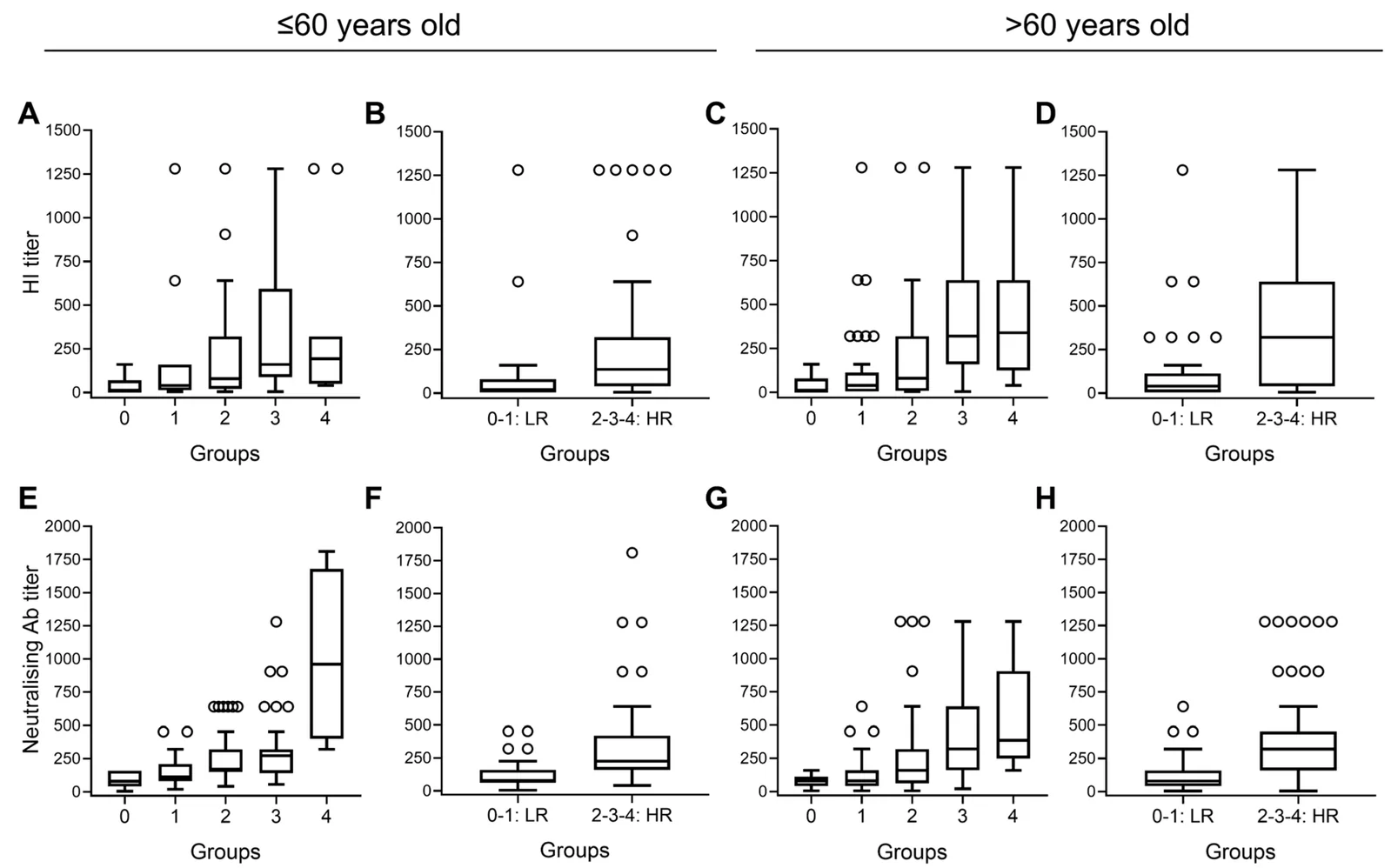
Stratification of LR and HR of subjects based on age. ((**A**,**B**), HI and (**E**,**F**) MN) The study population was stratified according to age, ≤60 and ((**C**,**D**), HI and (**G**,**H**), MN) >60 years old. Boxplots summarize antigen-specific observations obtained for the four vaccine strains. Individual dots correspond to antigen-specific observations (n = 300) and not to individual participants; dots outside the whiskers represent outliers.

To evaluate vaccine responsiveness while maintaining adequate numbers of participants within each responder category, subjects were re-stratified into two broader age groups: ≤60 years (n = 34) and >60 years (n = 41). This age dichotomization was applied exclusively for the responder analysis shown in Figure 5, as the original three-group classification (>25–<50, 50–65, ≥65 years) yielded subgroups with insufficient sample size after further stratification into high- and low-responder categories. Among those ≤60, 22 (65%) were female, and 12 (35%) were male; for those >60, 21 (51%) were female, and 20 (49%) were male. The median age was 53.5 years (range: 27–60 years; interquartile range: 42–57 years) and 75 years (range: 61–89 years; interquartile range: 68–78 years). As reported before applying, a mixed-effect model with ln of antibody titer as the outcome and independent variables for the LR vs. HR group, the antigen strain, and the baseline antibody titer in the population stratified as ≤60 years and >60 years, we found a significant difference between LR and HR (*p* < 0.001) in both groups. The differences were maintained by analyzing the MN titers for the individuals ≤60 (Figure 5E,F) and >60 (Figure 5G,H). These data indicate that the two groups of individuals that respond well or less efficiently to influenza vaccination can be distinguished both among younger and older adults. These data indicate that in addition to age, other factors contribute to the ability to develop an efficient response to vaccines.

A multiple logistic regression model where the low vs. high responder status was the dependent variable was applied as the outcome; the estimate suggested a possible association between age and low responsiveness. When adjusting for BMI, comorbidity, and frailty, age remained the variable most strongly associated with low responsiveness (adjusted OR = 1.45, 95% CI 0.98–2.14, *p* = 0.060); in other terms, for every 10-year increase in age, the adjusted odds of low responsiveness increased by 45% (*p* = 0.060). The other variables, BMI (OR = 1.04, 95% CI 0.91–1.19, *p* = 0.569), comorbidities (OR = 0.97, 95% CI 0.32–2.99, *p* = 0.963), and frailty (OR = 0.48, 95% CI 0.16–1.49, *p* = 0.203), were poorly associated with vaccine responsiveness in this sample. These findings indicate that the association with age observed in the study was not substantially affected by these potential confounders; overall, it should be considered that the statistical power of these findings relies on a small cohort.

### 3.5. Analysis of the Frequencies of Cells with Suppression Capacity in the High Responders and Low Responders to Influenza Vaccine

**Figure 6:**
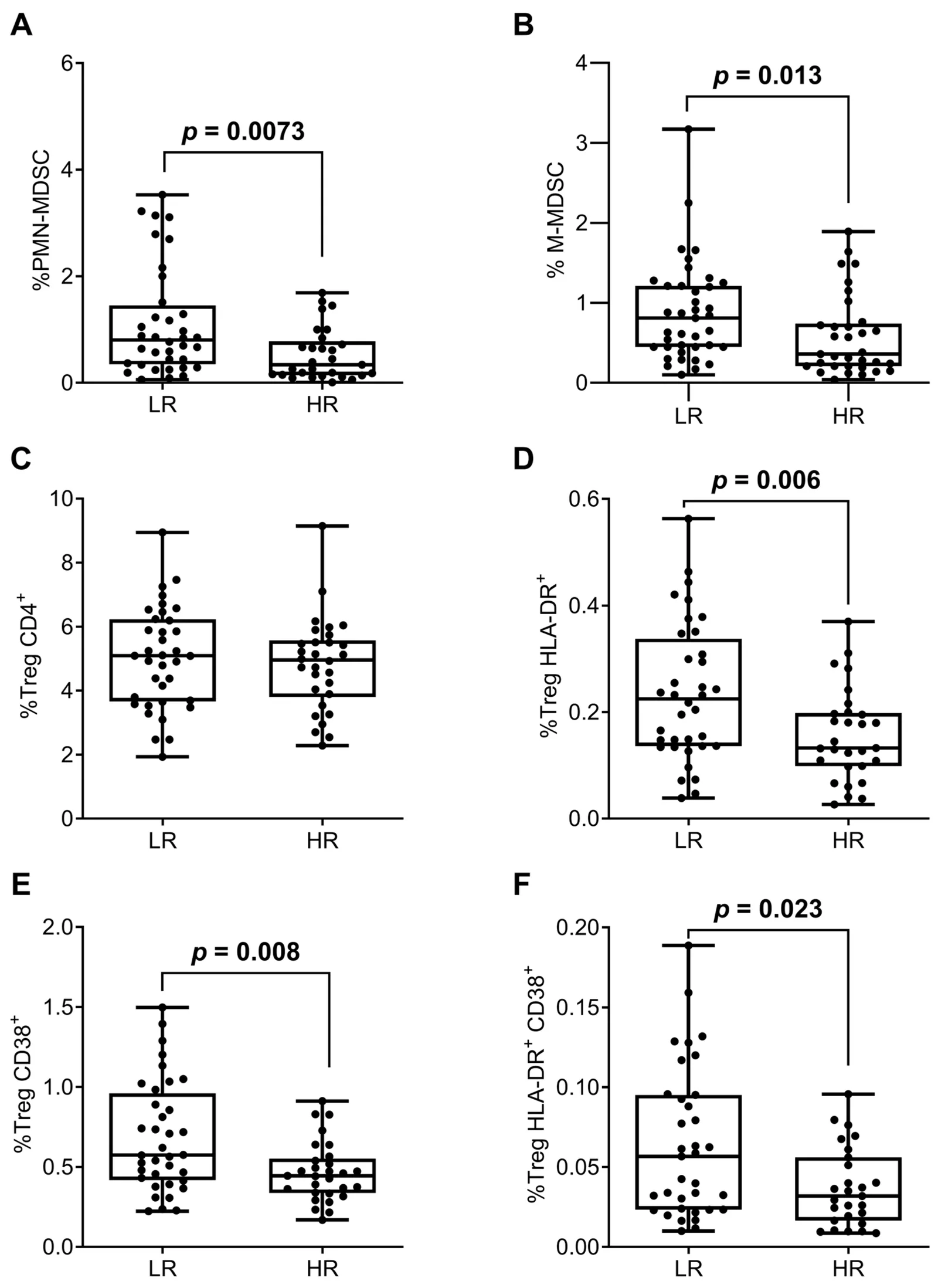
Percentages of suppressor cells in high and low responders to influenza vaccinations. The percentage of (**A**) PMN-MDSCs (n = 65) and (**B**) M-MDSCs (n = 72) in LR as compared with HR is shown. (**C**) The percentage of CD4^+^CD25^+^CD127^low^ Treg cells (n = 66), (**D**) the percentage of HLA-DR^+^ (n = 65), (**E**) CD38^+^ (n = 65), and (**F**) HLA-DR^+^CD38^+^ (n = 62) in LR and HR are shown. The non-parametric Mann–Whitney test was applied to evaluate the differences between groups.

To evaluate if the capacity to respond to the influenza vaccine had any association with the presence of cells with regulatory activity, the percentages of MDSCs and of Treg were evaluated in the HR and LR groups. The percentage of PMN-MDSCs and M-MDSCs were higher in the individuals who were less responsive to the vaccine (LR) as compared to the HR (0.81 (0.35–1.46) vs. 0.34 (0.15–0.78), *p* = 0.0073 and 0.81 (0.45–1.21 vs. 0.36 (0.21–0.74), *p* = 0.013 Figure 6A,B). Furthermore, although the percentages of CD4^+^CD25^+^ Treg were not different between LR and HR (Figure 6C), activated Treg including either the Treg expressing HLA-DR (0.22 (0.14–0.34 vs. 0.13 (0.1–0.2), *p* = 0.006) (Figure 6D) or the ones expressing CD38 (0.57 (0.42–0.96 vs. 0.44 (0.34–0.55), *p* = 0.008) (Figure 6E) or the cells expressing both activation markers (0.06 (0.02–0.1) vs. 0.03 (0.02–0.06), *p* = 0.023) (Figure 6F) were higher in the LR as compared to the percentages found in the HR. However, applying a multivariable logistic model, using LR status and age as the outcome and the BMI, comorbidity, and frailty as possible confounders, we did not confirm the significant differences between LR and HR for all immunoregulatory subsets, except for the CD4^+^CD25^+^ Treg that decreases with age (OR = 0.160, 95% CI 0.035–0.736, *p* = 0.019) and Treg CD38^+^ that increases with age (OR = 19.576, 95% CI 1.680–228.063, *p* = 0.018). However, the large OR and wide confidence interval for CD38^+^ Treg cells suggest interpreting this result as exploratory. Overall, these data suggest that there is a higher percentage of cells with regulatory activity, particularly Treg CD38^+^, which may be a potential marker associated with the capacity to respond to vaccinations.

### 3.6. Peripheral T Follicular Regulatory Helper Cells Increase with Age and Inversely Correlate with the Response to Influenza Vaccine

**Figure 7:**
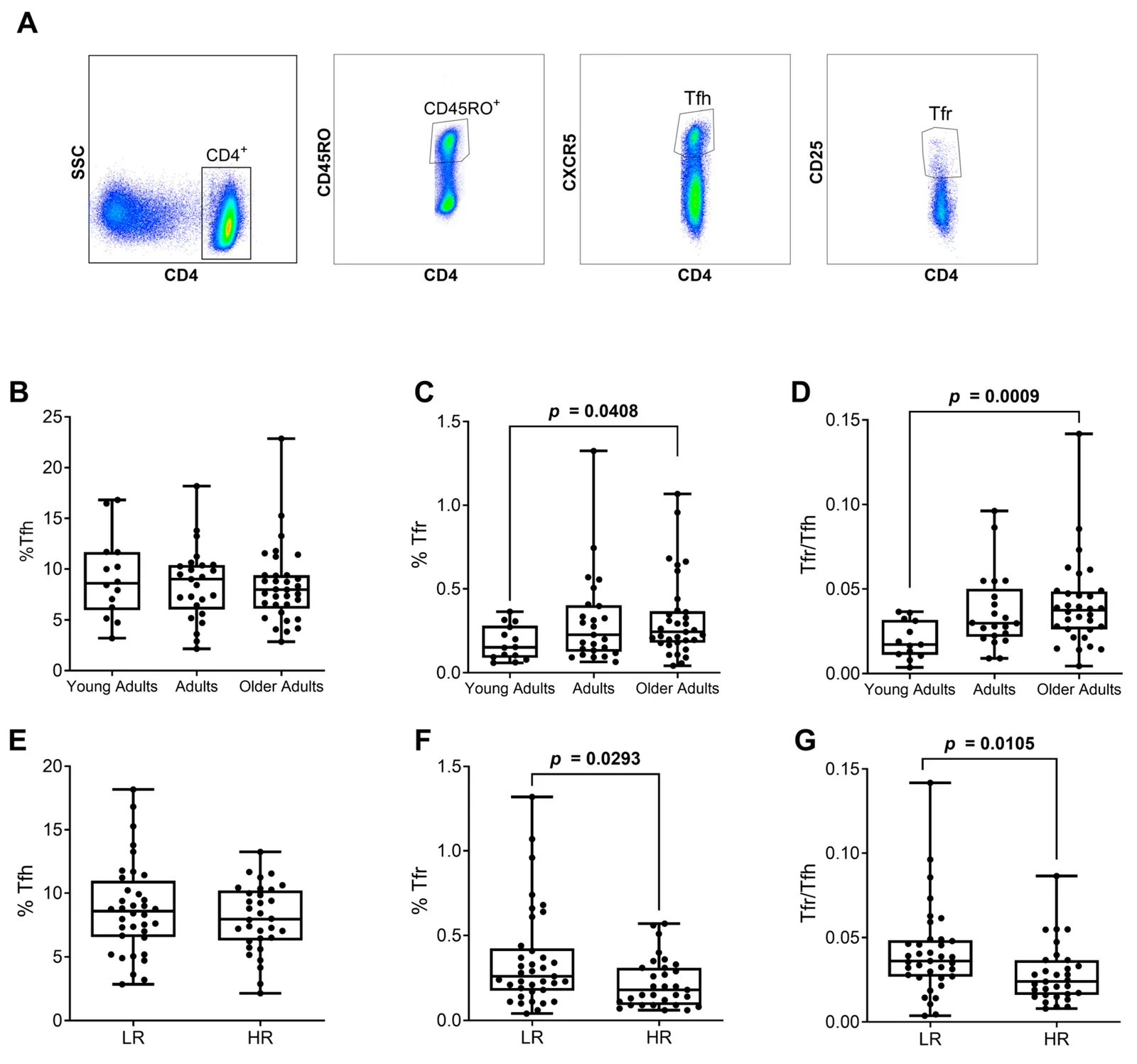
Peripheral T follicular regulatory helper cells increase with age and inversely correlate with the response to influenza vaccine. (**A**) Representative plots of the gating strategy are shown. (**B**) Percentage of CD4^+^ T follicular helper cells (Tfh) (n = 72), (**C**) percentage of T follicular regulatory helper cell (Tfr) (n = 72) and (**D**) *ratio* between Tfr and Tfh (Tfr/Tfh) (n = 68) in the older adults (≥65 years) as compared with the adults (≥50 and <65) and the young adults (>25 and <50) is shown. (**E**) Percentage of Tfh (n = 68), (**F**) Tfr (n = 68), and (**G**) the *ratio* between Tfr/Tfh in LR and HR are shown. The non-parametric Mann–Whitney test was applied to evaluate the differences between groups.

Antibody production and the generation of memory B cells are regulated by T follicular helper (Tfh) and T follicular regulatory (Tfr) cells in germinal centers [31,32]. Furthermore, there is also evidence that high frequencies of Tfh cells in peripheral blood predict sustained vaccine-induced titers of antibody response to vaccination [33,34]. Here, the percentages of Tfh cells and of Tfr cells were analyzed in the three age groups and in the LR and HR donors. Panel A of Figure 7 shows the gating strategy to identify Tfh and Tfr cells, which express CD4^+^CD45RO^+^CXCR5^+^ (Tfh) and CD4^+^CD45RO^+^CXCR5^+^CD25^+^ (Tfr) cells. The percentage of Tfh cells was not statistically different between the age groups nor among LR and HR (Figure 7B,E). However, significant differences were observed in the percentage of Tfr cells, which were higher in the older adults as compared to the young adults (0.24 (0.18–0.37) vs. 0.15 (0.09–0.28), *p* = 0.0408, Figure 7C) and lower in the HR as compared to the LR (0.18 (0.09–0.31) vs. 0.26 (0.18–0.43), *p* = 0.0293, Figure 7F). In addition, the *ratio* between Tfr cells and Tfh cells was higher in the older adults (0.04 (0.03–0.05) vs. 0.02 (0.01–0.03), *p* = 0.0009, Figure 7D) as compared to the young adults and lower in the HR as compared to the LR (0.02 (0.02–0.04) vs. 0.04 (0.03–0.05), *p* = 0.0105, Figure 7G). In addition, the association between age and the percentages of Tfh, Tfr, and Tfr/Tfh was assessed adjusting for BMI, comorbidity, and frailty (Tfh: β = −0.022, *p* = 0.528; Tfr: β = 0.004, *p* < 0.044; Tfr/Tfh: β = 0.001, *p* = 0.002). However, applying a multivariable logistic model, using LR status and age as the outcome and BMI, comorbidity, and frailty as possible confounders, we did not confirm the significant differences between LR and HR for these subsets. These data indicate that the presence of Tfr cells and the Tfr/Tfh cells correlate with age, and a larger study should be done to verify their association with the capacity to respond to flu vaccination. Altogether, the analysis of these cells and of their activation status could be useful candidate correlates of vaccine responsiveness.

## 4. Discussion

The variability in vaccine effectiveness among individuals is a common feature of different vaccines in both young and older individuals [1]. This study demonstrates that variability in the immune response to seasonal influenza vaccination is not solely determined by age but is associated with the presence of immunoregulatory cell populations. We observed a significant increase in MDSCs and activated Treg in older individuals, along with an inverse correlation between these populations and vaccine responsiveness. These findings suggest that immunoregulatory mechanisms, while essential for controlling chronic low-grade inflammation during aging, may also contribute to dampening the development of protective immunity following vaccination. The age-related accumulation of pro-inflammatory cytokines, commonly referred to as “inflammaging”, is a hallmark of immunosenescence and has been linked to impaired vaccine efficacy [4,35]. Our findings, which are in line with earlier findings in murine models [14,15] and in human studies [19,20], further demonstrate that both PMN-MDSCs and M-MDSCs increase progressively with age. The functional role of these cells in older individuals remains to be fully elucidated, but their expansion likely reflects an attempt to counterbalance chronic inflammation. Similarly, although the overall frequency of CD4^+^CD25^+^CD127^low^ Treg did not differ markedly between age groups, older participants exhibited a higher proportion of activated Treg expressing HLA-DR and CD38 markers. The expression of these activation markers has been reported to be associated with an enhanced suppressive capacity [36,37,38]. Therefore, this phenotype may represent an adaptive response to persistent inflammatory stimuli and simultaneously dampen vaccine-induced immunity. In humans, studies on age-related changes in regulatory T cells (Treg, CD4^+^CD25^+^CD127^low^) have yielded conflicting results [39]. Several reports indicate an increase in Treg frequency with age, both relative and absolute, often accompanied by a shift from naïve to effector/memory phenotypes [40,41,42]. In contrast, other investigations found no significant difference between young and older adults [43], or even a decline [44] and a diminished Th17/Treg *ratio* in very elderly cohorts [45]. These discrepancies likely reflect differences in studied age ranges, cohort composition, and phenotypic definitions of Treg, highlighting the need for standardized approaches to clarify how aging truly affects Treg homeostasis in the populations. Here, we found that the percentage of CD4^+^CD25^+^CD127^low^ Treg either did not differ between young adults (<50) and older adults (≥65) or was significantly lower in the older adult group as compared with the young adult group. However, by analyzing the expression of the activation markers HLA-DR and CD38 on the Treg, the older participants showed significantly higher percentages of activated Treg expressing HLA-DR or CD38 or both molecules as compared to the young adults. These data are in accordance with evidence that suggests that HLA-DR Treg increases with age in healthy donors [46]. Conversely, direct evidence of an age-related increase in the frequency of CD38 Treg is an original observation. The increased percentage of both MDSCs and activated Treg cells with high suppression capacity is consistent with the attempt by the immune system to contain inflammation in the elderly.

Considerable variability in vaccine-induced immunity is commonly observed at the population level, with individuals differing substantially in their capacity to mount effective antibody responses [47,48]. Although response to the influenza vaccine resulted in a significant increase in HI titers against each of the four vaccine strains, the magnitude of this response was not uniform across participants. Furthermore, the seroprotection at baseline against the four antigens included in the vaccine was observed in a high number of participants. This pre-existing immunity was not uniformly distributed among the strains examined, with the A/Brisbane strain showing the highest baseline protection rate (50%).

Overall, the percentage of protection after 28 days post-vaccination increases for all strains included in the vaccine, although to a varying extent. On the other hand, the percentage of seroconversion was low among the viral antigens except for the H3N2 A/Kansas, which reached about 70% of seroconversion. The high baseline titers could be due to previous exposure to the influenza virus or to earlier vaccinations [49,50]. However, baseline titers influence the responses to the influenza vaccination, with controversial results [51,52,53]. We observed in this cohort and described in depth that baseline titers correlate with a higher vaccine response. Due to the different baseline titers among donors, the identification of responders and non-responders following influenza vaccination is complex in both younger and older populations; therefore, participants were classified as LR and HR, according to the criteria described previously [30]. Notably, LR individuals displayed higher frequencies of MDSCs and activated Treg compared to HR, suggesting that immunoregulatory networks are associated with reduced vaccine responsiveness. Furthermore, although the percentage of Tfh cells, which regulate antibody production and the generation of memory B cells, was no different among the age groups nor among LR and HR; Tfr and the *ratio* between Tfr and Tfh was higher in the older group as compared to the young adult group and lower in the HR as compared to the LR, suggesting that excessive regulation may limit antibody production. These observations align with emerging evidence that baseline immune signatures, including Tfr/Tfh balance, can predict vaccine outcomes [54,55,56]. Although Tfh and Tfr are functional in the germinal center, there is evidence that baseline high frequencies of circulating Tfh cells predicted sustained vaccine-induced titers of antibody response to vaccination [57,58]. Nevertheless, applying a multivariable logistic model, adjusting for age, BMI, comorbidity, and frailty as possible confounders, we found that CD4^+^CD25^+^ Treg decreases with age and Treg CD38^+^ increases with age. However, we have to highlight that the cohort is small and a large OR and wide confidence interval for CD38^+^ Treg cells were observed; therefore, the results should be taken as exploratory, and further investigation could be useful to verify these findings in greater depth. Although the median-based classification for LR and HR is cohort-dependent and does not directly correspond to a recognized biological or clinical threshold, we used it for exploratory analysis and found associations with the frequency of immunoregulatory cells, as well as the same criteria applied in an independent cohort identified a different biological outcome [30]; however, these criteria require further investigation to strengthen their justification. Our findings highlight the potential of immunoregulatory markers, such as MDSC frequency, activated Treg phenotype, and Tfr/Tfh *ratio*, as potential candidates for vaccine responsiveness. Identifying individuals at risk of poor response could inform personalized vaccination strategies, including the use of adjuvants or targeted immunomodulation to enhance efficacy in vulnerable populations. This study has some limitations, including a relatively small sample size and the lack of functional assays to directly assess suppressive activity. Future research should validate these associations in larger cohorts and explore interventions aimed at modulating immunoregulatory pathways.

## 5. Conclusions

In this study, we identified an association between immunoregulatory populations and lower response to the seasonal anti-influenza vaccination. PMN-MDSCs, M-MDSCs, and activated T reg cells expressing either or both HLA-DR and CD38, and the regulatory subset of follicular T cells increased in older individuals after adjusting for possible confounders. Nevertheless, most immunoregulatory subsets did not remain independently associated with responder status after adjustment for possible confounders, except CD38^+^ Treg cells. These findings highlight that assessing immune characteristics prior to vaccination may offer insight into the factors that influence the effectiveness of vaccine-induced immunity in adults and older individuals. A better understanding of the immune signature associated with different response levels could facilitate the development of tailored vaccination strategies.

## Figures and Tables

**Table 1:** Sociodemographic characteristics of the study population.

Variable	Young Adults (n = 15)	Adults (n = 23)	Older Adults (n = 37)	Total (n = 75)
**Gender**				
Female	11 (73.3%)	14 (60.9%)	18 (48.6%)	43 (57.3%)
Male	4 (26.7%)	9 (39.1%)	19 (51.4%)	32 (42.7%)
**Age**				
Median	41	57	76	65
IQR	37.5–43	55–58.5	71–79	54–75.5
Range	27–49	52–65	66–89	27–89
**Education**				
Middle or lower	4 (26.7%)	6 (26.1%)	29 (78.4%)	39 (52.0%)
Higher	11 (73.3%)	17 (73.9%)	8 (21.6%)	36 (48.0%)
**Body mass index ^1^**				
Underweight	0 (0%)	0 (0%)	1 (2.9%)	1 (1.4%)
Healthy weight	12 (80.0%)	12 (52.2%)	13 (37.1%)	37 (50.7%)
Overweight	2 (13.3%)	8 (34.8%)	15 (42.9%)	25 (34.2%)
Obesity	1 (6.7%)	3 (13.0%)	6 (17.1%)	10 (13.7%)
**Smoking**				
Yes	2 (13.3%)	1 (4.3%)	7 (18.9%)	10 (13.3%)
No	13 (86.7%)	22 (95.7%)	30 (81.1%)	65 (86.7%)
**Frailty** ^2^				
Very fit/well (score 1–2)	14 (93.3%)	15 (65.2%)	20 (54.1%)	49 (65.3%)
Vulnerable-mildly frail (score >2)	1 (6.7%)	8 (34.8%)	17 (45.9%)	26 (34.7%)
**Score H test** ^3^				
≤23 (F); ≤36 (M)	1 (8.3%)	8 (36.4%)	14 (51.9%)	23 (37.7%)
>23 (F); >36 (M)	11 (91.7%)	14 (63.6%)	13 (48.1%)	38 (62.3%)
**Medical conditions** ^4^				
Yes	5 (33.3%)	9 (39.1%)	29 (78.4%)	43 (57.3%)
No	10 (66.7%)	14 (60.9%)	8 (21.6%)	32 (42.7%)
**Chronic cardiac diseases**				
Yes	2 (13.3%)	8 (34.8%)	24 (64.9%)	34 (45.3%)
No	13 (86.7%)	15 (65.2%)	13 (35.1%)	41 (54.7%)
**Diabetes**				
Yes	1 (6.7%)	1 (6.7%)	7 (18.9%)	9 (12%)
No	14 (93.3%)	22 (95.7%)	30 (81.1%)	66 (88%)
**Asthma**				
Yes	2 (13.3%)	2 (8.7%)	4 (10.8%)	8 (10.7%)
No	13 (86.7%)	21 (91.3%)	33 (89.2%)	67 (89.3%)
**COPD**				
Yes	-	1 (4.3%)	6 (16.2%)	7 (9.3%)
No	15 (100.0%)	22 (95.7%)	31 (83.8%)	68 (90.7%)

^1^ data available for 72 participants out of 75. ^2^ frailty assessed using the Clinical Frailty Scale. ^3^ handgrip strength thresholds: ≤23 kg (female) and ≤36 kg (male). Data available for 61 participants out of 75. ^4^ includes chronic conditions (e.g., hypertension, diabetes).

## Data Availability

The data presented in this study are available upon request from the corresponding author. Due to the ongoing nature of this study, the data are not publicly available currently.

## Abbreviations

The following abbreviations are used in this manuscript:

MDSCs	Myeloid-derived suppressor cells
Treg	T regulatory lymphocytes
Tfr	T follicular regulatory cells
Tfh	T follicular helper cells
QIV	Quadrivalent influenza vaccine
HI	Hemagglutination inhibition
MN	Micro-neutralization
